# A deep neural network for adaptive spatial smoothing of task fMRI data

**DOI:** 10.3389/fnimg.2025.1554769

**Published:** 2025-04-29

**Authors:** Zhengshi Yang, Xiaowei Zhuang, Mark J. Lowe, Dietmar Cordes

**Affiliations:** ^1^Cleveland Clinic Lou Ruvo Center for Brain Health, Las Vegas, NV, United States; ^2^Imaging Institute, Lerner Research Institute, Cleveland Clinic, Cleveland, OH, United States; ^3^Department of Psychology and Neuroscience, University of Colorado, Boulder, CO, United States

**Keywords:** functional MRI, adaptive spatial smoothing, deep neural network, brain activation, task fMRI analysis

## Abstract

Over the past decade, functional magnetic resonance imaging (fMRI) has emerged as a widely adopted *in vivo* imaging technique for examining neural activity in the brain. A common preprocessing step in fMRI analysis is spatial smoothing, which helps in detecting cluster-like active regions. The use of a heuristically selected Gaussian filter for spatial smoothing is frequently preferred due to its simplicity and computational efficiency. Neurons in the cerebral cortex are located within a thin sheet of gray matter at the surface of the brain, and the human brain’s gyrification results in a complex gray matter anatomy. For task-based fMRI activation analysis, isotropic Gaussian smoothing can reduce spatial specificity, introducing spatial blurring artifacts where inactive voxels near active regions are mistakenly identified as active. This blurring is beneficial for group-level analysis as it helps mitigate anatomical variability across subjects and inaccuracies in spatial normalization. However, it poses challenges in subject-level analysis, particularly in clinical applications such as presurgical planning and fMRI fingerprinting, which demand high spatial specificity. Previous studies have proposed several adaptive spatial smoothing techniques to address these issues. In this study, we introduce a versatile deep neural network (DNN) that builds on the strengths of previous approaches while overcoming their limitations. This method can incorporate additional neighboring voxels for estimating optimal spatial smoothing without significantly increasing computational costs, making it suitable for ultrahigh-resolution (sub-millimeter) task fMRI data. Furthermore, the proposed neural network incorporates brain tissue properties, enabling more accurate characterization of brain activation at the individual level.

## Introduction

Blood oxygen level-dependent (BOLD) functional magnetic resonance imaging (fMRI) is an imaging technique developed to capture time-varying changes in deoxyhemoglobin concentration within the brain (Arthurs and Boniface, 2002). Unlike resting-state fMRI, which examines spontaneous signal synchronicity across brain regions without requiring active tasks from participants, task fMRI requires participants to engage in specific tasks, making it valuable for localizing brain regions involved in certain functions.

Since its introduction in the 1990s, fMRI has become a prominent tool in cognitive neuroscience, clinical psychology, and surgical planning due to its non-invasive nature, high spatial resolution, and reasonable temporal resolution. However, the BOLD signal in fMRI time series is often contaminated with both structured and unstructured noise from various sources (Liu, 2016). Moreover, the BOLD contrast in higher-order cognitive tasks can be quite small—less than 1%—which diminishes the sensitivity and reliability of fMRI during statistical analyses (Huettel et al., 2004). To address this, spatial smoothing is commonly used as a preprocessing step to enhance the signal-to-noise ratio (SNR) of the data. The Gaussian filter, with a heuristically chosen full-width at half maximum (FWHM), remains the dominant method for smoothing fMRI data due to its simplicity and computational efficiency. Gaussian smoothing is universally applied to all voxels to derive smoothed time series, and then a univariate general linear model (GLM) is carried out for fMRI activation analysis. Spatial smoothing can be seen as a low-pass spatial filtering process that removes high-frequency components. This technique is effective in suppressing thermal noise and improving sensitivity to detecting BOLD signals, facilitating the identification of active regions (Hartvig and Jensen, 2000).

The variability in cortical gyrification (the pattern and degree of cortical folding) between subjects and imperfections in spatial normalization from individual to template space are the hurdles compromising group analysis; Gaussian smoothing facilitates the analysis at the cost of compromised spatial specificity at the individual level. Considering that the cortex is highly folded with variable granularity of activation profiles (i.e., spatial patterns, orientations, and contrast-to-noise ratio), applying a fixed isotropic Gaussian filter inevitably dilates active regions and leads to false active voxels at the subject level (Kamitani and Sawahata, 2010). This limitation is particularly problematic for applications where activation at the subject level is of particular interest, such as surgical planning (Silva et al., 2018) (i.e., estimate the location of cortical areas involved in speech and language (Signorelli et al., 2001; Bizzi et al., 2008) in relationship to brain tumors for safe resection) and fMRI fingerprinting, although current fMRI fingerprinting studies are mainly focused on resting state data due to its wider accessibility (Finn et al., 2015).

To address these challenges, several adaptive spatial smoothing methods have been proposed (Cordes et al., 2012; Zhuang et al., 2017; Yang et al., 2018). These methods aim to improve spatial specificity at the subject level by tailoring the smoothing parameters for each voxel based on the time series of surrounding voxels. The multivariate extension of GLM, such as canonical correlation analysis (CCA) (Zhuang et al., 2020), has been developed for this purpose. CCA is applied to derive a set of coefficients to maximize the correlation between the task design matrix and the unsmoothed time series from a voxel and its neighboring voxels. The optimized coefficients of unsmoothed time series are treated as the contribution of neighboring voxels in deriving smoothed time series of the center voxel, and the coefficients of the task design matrix represented the estimated activity of the center voxel under the task conditions (Friman et al., 2003). Conventional CCA can be solved efficiently with an analytical solution. Since CCA was introduced for fMRI activation analysis in 2001 (Friman et al., 2001), it was soon recognized that a constraint on neighboring time series is required to alleviate the spatial blurring artifact induced by the extra degree of freedom. To address this issue, multiple constraints on the weights of the neighboring voxels were introduced in previous studies (Cordes et al., 2012; Zhuang et al., 2017; Yang et al., 2018). However, these constraints either eliminate the analytical solution for CCA or make it dependent on an exponentially increasing number of subproblems (e.g., 2^26 sub-problems for 3 × 3 × 3 neighboring voxels), leading to longer computation times than the numerical solution (Cordes et al., 2012; Zhuang et al., 2017). Including more neighboring voxels in the analysis leads to substantially increased computational time cost; therefore, the constrained CCA was initially proposed to include only the nearest neighboring voxels in the same slice (3 × 3) (Cordes et al., 2012), and it was later extended to include voxels from neighboring slices with a more efficient optimization algorithm (Yang et al., 2018). Alternatively, the original time series can be anisotropically smoothed by a set of pre-specified oriented filters, and then a constrained CCA is applied to determine the optimal smoothing orientation, but not the shape, of each voxel (Yang et al., 2018), although the constraint applied to these filters compromises their ability to fit an arbitrary orientation (Rydell et al., 2006).

The technical development of fMRI acquisition has improved the contrast-to-noise ratio and made it feasible to collect meaningful data with higher spatial resolution. In contrast to the typical voxel size of 3 × 3 × 3 mm^3^ in early fMRI studies, smaller voxel sizes at the millimeter level or even submillimeter level (Huber et al., 2020; Lawrence et al., 2019) are more commonly used. The higher spatial resolution allows cognitive neuroscientists to detect the subtle activity difference between anatomically close regions or subregions in complex tasks (Yassa and Stark, 2011). Since the BOLD signal in smaller voxels is weaker than the signal in larger voxels due to less deoxyhemoglobin in smaller voxels, spatial smoothing remains necessary for most fMRI studies. In addition, including more neighboring voxels for adaptive fMRI smoothing is beneficial due to the finer resolution of the data. However, considering more neighboring voxels substantially increases the computational cost of these constrained CCA methods (Yang et al., 2018).

In this study, we aim to develop a deep neural network (DNN) model to adaptively estimate the optimal spatial smoothing for the task fMRI data at the subject level. By deepening the convolutional layers in the model, more neighboring voxels are taken into consideration in a time-efficient manner. In addition, instead of pre-specifying a set of filters, the filters in the model are estimated from the data itself, which could fit any shape as demonstrated in natural and medical image processing (Garcia-Garcia et al., 2017; Razzak et al., 2018). We hypothesize that the proposed method provides a more accurate estimation of brain activation.

## Methods

### Brief overview of GLM and CCA

GLM is the most commonly used approach to detect brain activation in task fMRI data, which is formulated as


(1) y=Xβ+∈

$y\in {ℛ}^{T\times 1}$ represents the time series of a voxel, which is usually spatially smoothed with a heuristically selected Gaussian filter instead of the original unsmoothed time series. $X\in {ℛ}^{T\times N}$ is the design matrix modelling the BOLD response stimulated by the N types of stimuli in the task, which is computed by convolving binary task design with the canonical hemodynamic response function. T is the number of fMRI volumes. The coefficient vector $\beta =\left(\beta_{1},\ldots ,\beta_{N}\right)\in {ℛ}^{N\times 1}$ in Equation 1 is estimated by linear regression such that the Frobenius norm of the residual term ∈=y−Xβ is minimized, which is equivalent to maximizing the correlation ρ between y and Xβ , namely $\max_{\beta }\rho \left(y,X\beta \right)$ . Each element of the coefficient vector β represents the activity strength of the voxel corresponding to the task stimulus, and ρ indicates how strongly a voxel is relevant to the task across all task stimuli modeled in the design matrix X .

Spatial smoothing can be treated as a weighted summation of the time series from a voxel and its neighboring voxels, formulated as $y\text{≡}Y\alpha =\sum_{i}y_{i}\alpha_{i}$ . For Gaussian smoothing, the spatial weight coefficient α is a constant vector across all voxels, and its value is solely determined by the FHWM. As to the aforementioned CCA methods, α is estimated together with β by maximizing the correlation


(2) $$\max_{\alpha ,\beta }\rho \left(y,X\beta \right),\mathrm{where}\;y\text{≡}Y\alpha$$

where Y is comprised of unsmoothed time series from a certain voxel and its neighboring voxels. The CCA method formulated in Equation 2 can be treated as the multivariate extension of the univariate GLM model (Thompson, 2005). Y is of the dimension T×9 when only the in-slice 2D 3×3 nearest neighboring voxels are considered (Friman et al., 2003), and it is of the dimension T×27 when 3D 3×3×3 neighboring voxels are included (Yang et al., 2018). The activity estimated from the equation is assigned to the center voxel. If the original fMRI data is first anisotropically smoothed by a set of filters (Yang et al., 2018), and then the filtered time series are fed into Equation 2. In this case, each element in α represents the weight of individual filters for the center voxel. While CCA can derive a voxel-specific spatial smoothing strategy, it is prone to spatial blurring artifacts due to the extra degree of freedom unless proper constraints are applied to the weight coefficient α .

When CCA was applied to fMRI activation analysis, a non-negative constraint on α was first proposed and was solved analytically (Friman et al., 2003), and then the later proposed constraints were solved analytically (Cordes et al., 2012) or numerically (Zhuang et al., 2017; Yang et al., 2018). When the in-plane 3×3 nearest neighboring voxels were considered in the analysis, the sum constraint was illustrated to have the best performance (Zhuang et al., 2017), where the weight of the center voxel was no less than the sum of the weights of all neighboring voxels, and the weights of all voxels were non-negative. Although CCA with a sum constraint on 3×3×3 neighboring voxels can still be solved with reasonable time cost, further increasing neighboring voxels leads to a substantially increased computational burden (Yang et al., 2018). Given these challenges, we propose a deep neural network (DNN) that utilizes the advantages of CCA while improving computational efficiency and scalability. Instead of using predefined filters, our DNN model learns spatial filters from the data, thus allowing for more flexible smoothing strategies. The convolutional layers in the model capture spatial information from neighboring voxels, while the fully connected layers optimize the weights of the smoothed time series to improve activation detection.

### Architecture of DNN

**Figure 1:**
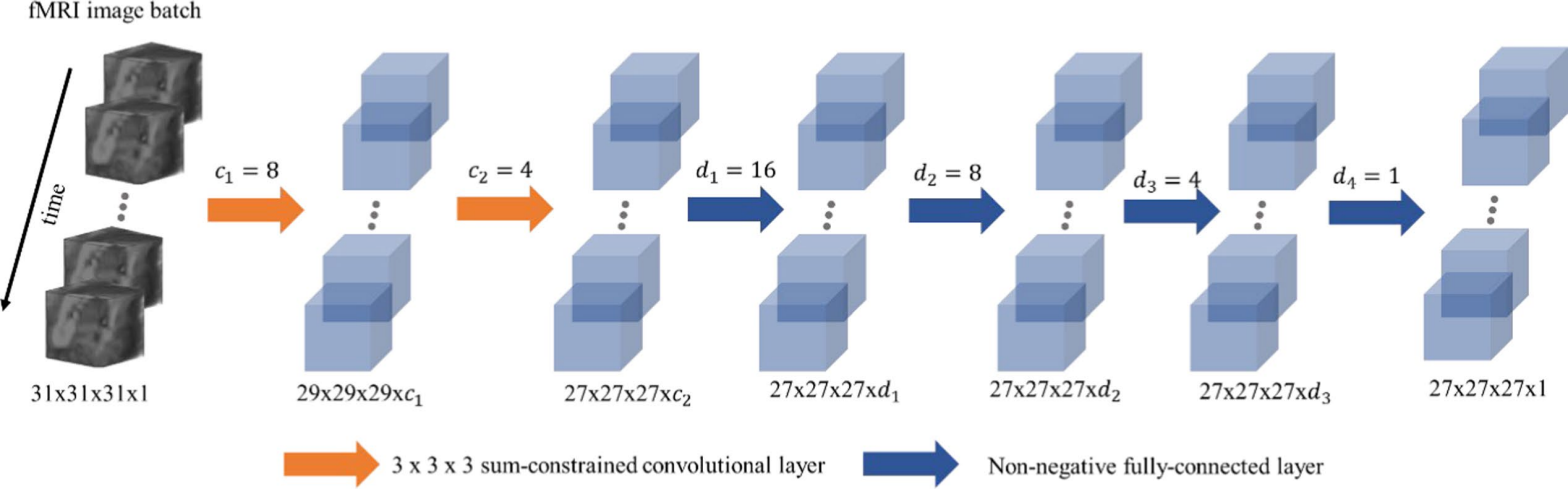
Architecture of the proposed deep neural network for adaptive smoothing. The 3D convolutional layers are applied to the spatial space. The fully connected layers are applied on the final output channel from the last convolutional layer. A sum constraint is applied on the convolutional layers and a non-negative constraint is applied to the fully connected layers.

The DNN model consists of multiple 3D convolutional layers followed by fully connected layers, as illustrated in Figure 1. The model is trained using unsmoothed fMRI data and outputs the DNN-smoothed time series. The 3D convolutional layers act as the data-driven spatial filters learnt from the data itself, allowing for flexible adaptation to various data characteristics. In contrast, the shapes and orientations of the anisotropic filters used in previous studies are pre-specified (Yang et al., 2018). The fully connected layers in the DNN model assign weights to the smoothed time series from the convolutional layers, ensuring that the model produces an optimized smoothed time series.

To reduce the memory load, fMRI data are partitioned into smaller batches through a data generator and then fed to the model with the shape of n×T×x×y×z×1 , where n is the batch size, T is the length of fMRI time points, 1 is the number of channels, and x×y×z (31 × 31 × 31 in this study) is the 3D spatial dimension of the cropped fMRI data.

The 3D convolutional layers have a kernel size of 3 × 3 × 3, and the output time series from each filter are assigned to the center voxel. In this way, the output time series from a convolutional layer contains the information from the nearest neighboring voxels. By stacking an extra convolutional layer, the information from the second nearest neighboring voxels can propagate to the center voxel and so forth. Compared to a convolutional layer with a larger kernel size, stacking multiple layers with smaller kernel sizes has the advantage of computational efficiency (Yang et al., 2020a). Let $\omega_{i}\in {ℛ}^{F_{i-1}\times 3\times 3\times 3\times F_{i}}$ denote the weight of the filters in one convolutional layer, where $F_{i}$ represents the number of filters in the i -th convolutional layer and $F_{i-1}$ is the number of filters in the previous layer. For the first convolutional layer in the model ( i=1 ), $F_{i-1}$ equals to the number of channels of input fMRI data, namely $F_{0}=1$ . Similar to the conventional CCA requiring constraints on the neighboring voxels to reduce spatial blurring artifacts, constraints are applied on the convolutional layers. Considering that CCA with a sum constraint has the best performance for fMRI activation analysis (Zhuang et al., 2017), the center element of the kernel is required to be no less than the summation of the rest 26 elements, and all elements in the kernel are non-negative, which is formulated as


$$\omega_{i}\left(f_{i-1},2,2,2,f_{i}\right)\geq \underset{\begin{array}{c} m,n,q\;\\ \mathrm{except}\;m=n=q=2 \end{array}}{\sum }\omega_{i}\left(f_{i-1},m,n,q,f_{i}\right)\text{,}$$

$$f_{i-1}=\left\{1,\ldots ,F_{i-1}\right\},f_{i}=\left\{1,\ldots ,F_{i}\right\}$$

$$\mathrm{and}\;\omega_{i}\left(f_{i-1},m,n,q,f_{i}\right)\geq 0.$$

The fully connected layers are then applied to determine the weights of the spatially filtered time series from the last convolutional layers. The last fully connected layer has only one channel, and its output is the final time series after DNN spatial smoothing. The weights in the fully connected layers are required to be non-negative. The non-negative constraint in the fully connected layers is used for two reasons. First, the DNN model should not artificially flip the sign of the time series, which can lead to the risk of erroneously identifying an activated ( β>0) region as deactivated ( β<0 ) and vice versa. Second, spatial smoothing is intended to work as a low-pass spatial filter to improve the SNR, having a negative weight on certain spatial filters no longer preserves this property and it may exacerbate the following statistical analysis.

The DNN model is optimized with a customized cost function. Identifying robust brain activity in regions involved in the task is a crucial step in analyzing task fMRI data; therefore, the proposed DNN model should better suppress the noise in the data and have stronger statistical power to identify active brain regions. At the same time, the brain activity in the regions not involved in the task should not be overestimated, leading to lower spatial specificity with more false active voxels. When the neuron cells in the gray matter are engaged in the task, it causes an increased BOLD signal, which is detected by fMRI. Considering that most of the brain activity, if not all, is expected to occur within the gray matter, the cost function for the DNN model is defined as the ratio of brain activity strength between gray matter (GM) and non-gray matter (non-GM; including both white matter and cerebrospinal fluid), which is formulated as


$$ℒ\left(Y,X\right)=-\frac{\bar{r}\left(Y_{GM},X\right)}{\bar{r}\left(Y_{non-GM},X\right)}\text{.}$$

The brain activity strength is characterized by the average correlation $\bar{r}$ from GLM analysis between DNN-output time series ( Y )and task design matrix ( X ) in each iteration. The brain tissue mask is generated from T1 structural MRI data and then coregistered to fMRI space. The non-GM mask is eroded twice to avoid the contribution of gray matter due to the partial volume effect. Considering that not all regions in the gray matter are active during the task, there is a concern that no penalty on these inactive GM regions in the cost function could lead to false active regions. However, if brain activity is “artificially” induced in inactive GM regions, the same phenomenon is expected to occur within the non-GM tissue since the same model is used across all voxels regardless of their brain tissue property. Therefore, the cost function could indirectly suppress false active regions in GM tissue. A few recent studies argued that white matter might contain functional signals (Schilling et al., 2023; Courtemanche et al., 2018). However, its hemodynamic response function was distinct from the canonical hemodynamic response function, and the magnitude was substantially lower than in gray matter (Li et al., 2019). Therefore, the proposed cost function remains valid for activation detection in gray matter.

### MRI data

The structural and functional data used in the study were obtained from the Human Connectome Project (HCP) Young Adult (https://www.humanconnectome.org/study/hcp-young-adult). The 3 T MRI imaging data from 88 subjects were used in this study. All subjects were males with ages in the range of 26–30 years, having complete T1, resting-state fMRI, and task fMRI scans. The demographic criteria were heuristically selected to have a proper sample size for comparing the methods used in the study. The structural T1 images were acquired with a resolution of 260 × 311 × 260 to yield 0.7 mm × 0.7 mm × 0.7 mm isotropic voxel size. The working memory task fMRI data were acquired with 405 time points from a gradient-echo fast EPI sequence with these parameters: multiband factor 8, TR/TE = 720/33.1 ms; flip angle = 52 degrees; 72 slices; spatial resolution = 2 mm × 2 mm × 2 mm and imaging matrix = 104 × 90. The resting-state fMRI data were acquired with an identical pulse sequence except with 1,200 time points.

The first 15 volumes of working memory fMRI data were discarded to avoid data with unsaturated T1 signals. Minimally preprocessed fMRI data (in standard MNI space) (Glasser et al., 2013) after an additional linear detrending step to remove signal intensity drift (Tanabe et al., 2002) were treated as raw fMRI data in our analysis. Regressors modeling movement or physiological noise were not used in the processing steps since including these regressors did not show any evidence of improvement with the HCP data (Barch et al., 2013). The task itself represented an event-related task design consisting of targets, non-targets, and lure conditions. Within each working memory task fMRI run, four different stimulus types, including places, tools, faces, and body parts, were presented in separate blocks. ½ of the blocks used a 2-back working memory task and ½ used a 0-back working memory task for each run. Further details of the HCP 3 T MRI protocols and task designs can be found in (Barch et al., 2013). The task design matrix $X\in {ℛ}^{T\times 3}$ was computed by convolving binary task conditions with the canonical hemodynamic response function.

### Simulation

A set of 20 simulated task fMRI sessions was generated with resting-state fMRI data from 20 randomly selected subjects, together with the working memory task design matrix, to mimic real fMRI data for evaluating the performance of different denoising techniques. The resting-state fMRI data were truncated to match the length of task fMRI data and then wavelet-resampled in the time domain to keep the same spatial arrangement (Zhuang et al., 2020), denoted as $y_{r}$ , which was treated as the signal irrelevant to the task. The gray matter voxels within six bilateral regions in the AAL atlas (Tzourio-Mazoyer et al., 2002), including the anterior cingulate cortex, precentral gyrus, inferior frontal gyrus, insula, middle frontal gyrus, and middle temporal gyrus, were simulated to be active. Each region was specified with one certain weight vector *β* for the task conditions. The signal of an active voxel was computed as $s=X\left(\beta +\in \right)$ , where ϵ was a random vector matching the size of *β* with value ranging from −0.1 to 0.1. For an active voxel, the simulated time series was generated as $y=y_{r}+f*s$ , where f was a constant value across all active voxels. For inactive voxels, $y_{r}$ was the simulated time series for each voxel, namely $y=y_{r}$ . In this study, the weight vector *β* was defined with three situations covered: (1) only involved in one task condition, e.g., [1 0 0], [0 1 0], and [0 0 1]; (2) involved in two conditions, e.g., [0.3 1 0] and [0.45 0 0.95]; and involved in all three conditions, e.g., [1 0.3 0.3].

### Analysis

With both simulated and real fMRI data, multiple analysis methods were carried out to derive the correlation between fMRI time series and task design matrix, including GLM without spatial smoothing, GLM with full width at half maximum (FWHM) = 6 mm Gaussian smoothing (S6GLM), conventional CCA without a constraint, CCA with a sum constraint (sumCCA), and the proposed DNN method. The computation was run on a Xi computer (https://www.xicomputer.com/) with 2 × Intel Xeon Silver 4,208 processors (32 cores in total) and 2 × NVIDIA GeForce RTX 3090 graphical cards. In-house MATLAB (The MathWorks, Inc., version R2022a) scripts were used to run GLM, S6GLM, CCA, and sumCCA. GLM and S6GLM took approximately 80 s per subject without parallel computing. Following previous studies (Zhuang et al., 2017; Yang et al., 2018), CCA and sumCCA were run with the nearest neighboring voxels considered, namely 3 × 3 × 3 neighboring voxels. Parallel computing with 24 cores was enabled for CCA and sumCCA. It took 2 min to run a conventional CCA and 15 min to run sumCCA per subject. DNN was conducted with two 3D conventional layers, which was equivalent to 5 × 5 × 5 neighboring voxels. TensorFlow 2.11.1 (https://www.tensorflow.org/) was used to run the DNN model. It took approximately 10 min per subject to train the DNN model on a single graphical card. Note that it took approximately 70 min per subject for sumCCA if 5 × 5 × 5 neighboring voxels were considered.

With the known activation status of each voxel in the simulated data, a receiver operating characteristic curve (ROC) was used to evaluate the overall sensitivity and specificity of each analysis method. The area under the ROC curve (AUC) was computed with a limited false-positive rate (FPR) range of 0–0.1 instead of a full range of 0–1. Considering that inactive voxels were usually strictly controlled in fMRI analysis, the AUC value computed with a limited FPR range was more meaningful than the value obtained with the full FPR range.

With the real fMRI data from 88 subjects, we first used a 2D histogram to evaluate the relationship of the correlation values obtained from voxel-wise GLM analysis and methods. The correlation value could be treated as the estimated strength of how strongly a voxel was involved in the task. The 2D histogram could help visualize how different methods estimate activation across a range of voxel correlations, from low to high, in the GLM. With the hypothesis that low-correlation voxels in GLM were less likely to be active and high-correlation voxels in GLM were more likely to be active, an approach that consolidated the strength of active voxels and had less or no influence on inactive voxels could help better discriminate active voxels from inactive voxels.

Because parametric statistic tests (e.g., t statistics) were not applicable to constrained CCA (Zhuang et al., 2017), a non-parametric permutation test was used to compare different methods. Resampling fMRI data and then replicating the analysis as with original data were used to generate a null distribution and then determine the significance level in fMRI analysis. In this study, we took wavelet-resampled resting-state fMRI data and then ran analysis with different analysis methods. To reduce computational time, only one set of resampled data was generated for each subject. The distribution of correlation from null data was computed separately for each subject, and then the correlation value at the 99.9 percentile, denoted as $r_{p}$ , was recorded, which approximated the magnitude at the significance level of *p* = 0.001 for each method. This value could be treated as an assessment of how the spatial smoothing strategy influenced the null data, which was important for evaluating if a time-consuming non-parametric permutation test was needed to determine the statistical significance of the activation map. More importantly, the magnitude difference of $r_{p}$ between one certain spatial smoothing method and GLM without spatial smoothing, namely $\Delta r_{p}$ , reflected how strongly the smoothing strategy exaggerated the correlation of inactive voxels.

## Results

### Simulated data

**Figure 2:**
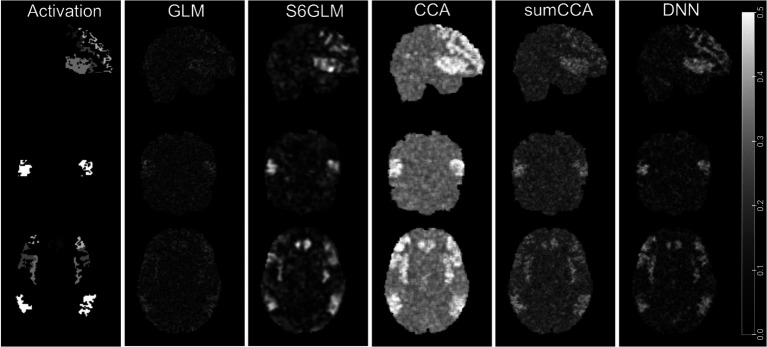
Simulated activation patterns and the corresponding correlation maps derived from analysis methods, including GLM without spatial smoothing, GLM with FWHM = 6 mm Gaussian smoothing, conventional CCA, CCA with a sum constraint (sumCCA), and the proposed DNN method.

**Figure 3:**
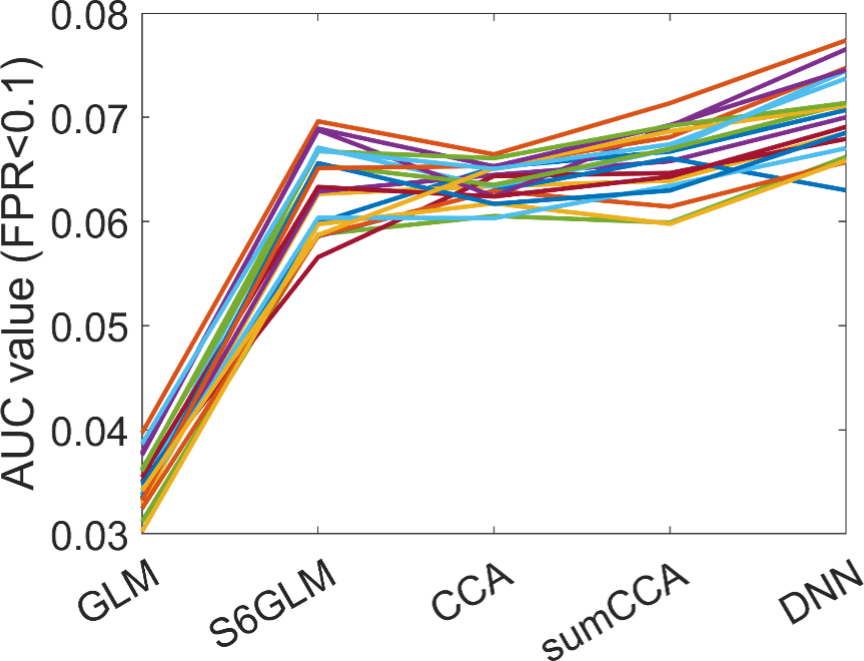
AUC values from different methods for 20 simulated fMRI sessions.

The correlation map from a single simulated fMRI session is shown in Figure 2, together with the ground truth activation map. Without spatial smoothing, the active regions in the GLM-derived spatial map appeared to be obsolete and barely differentiated from inactive voxels. In a comparison of S6GLM vs. GLM, Gaussian smoothing significantly enhanced the activation map, making the active regions more distinguishable. Similarly, the active regions derived from conventional CCA were also more discriminative compared to GLM without spatial smoothing. At the same time, CCA had higher correlation values across the entire brain than other methods regardless of the activation status; such a phenomenon was alleviated when the sum constraint was implemented. The proposed DNN method had a comparable correlation magnitude with GLM in the inactive regions, but it provided better contrast between inactive regions and active regions. The mean AUC values in the FPR < =0.1 range (max value 0.1) were 0.035, 0.063, 0.064, 0.066, and 0.070 for GLM without spatial smoothing, S6GLM, conventional CCA, sumCCA, and DNN, respectively. The AUC values calculated from all 20 simulated fMRI sessions are shown in Figure 3.

### Real data

**Figure 4:**
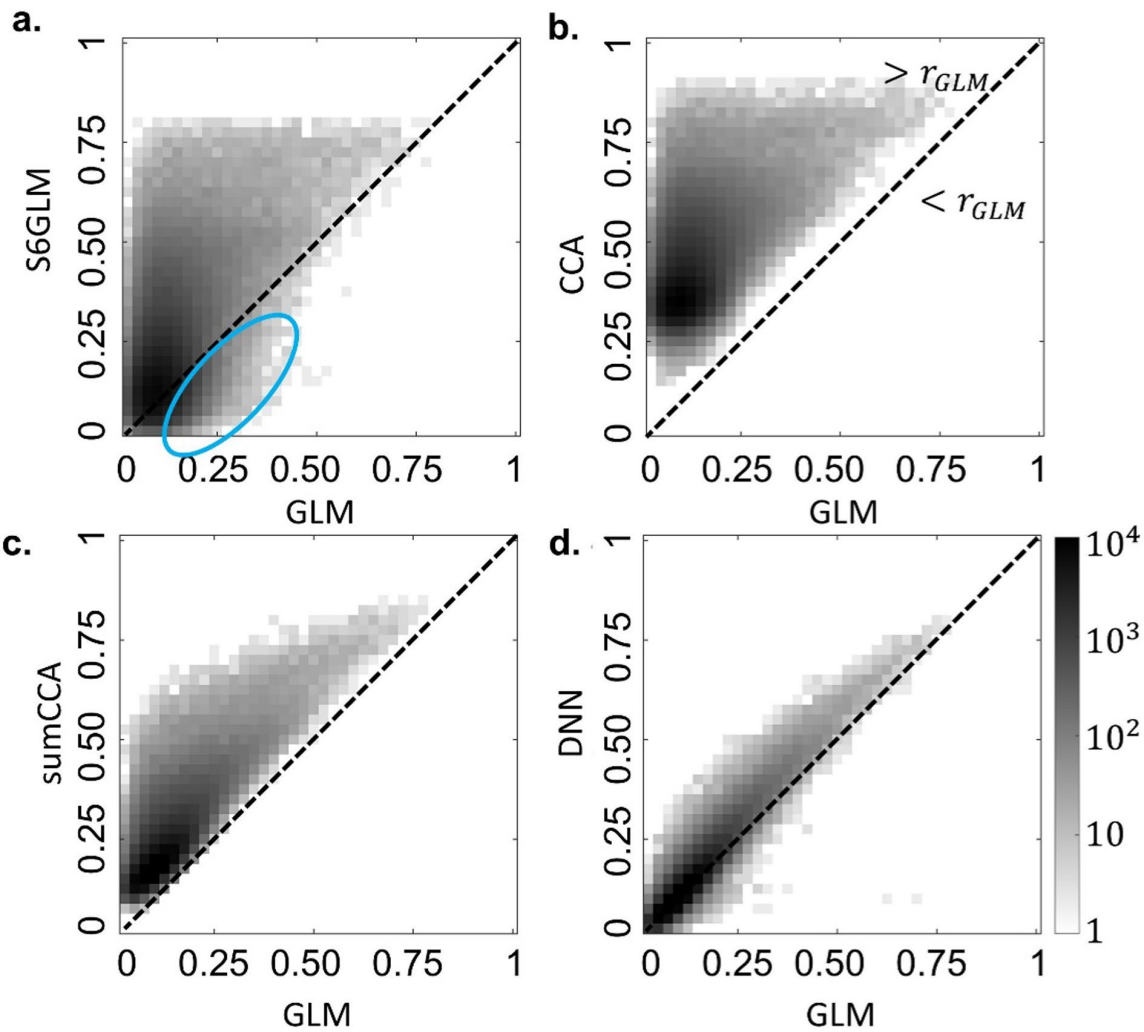
2D histograms of correlations obtained from GLM without spatial smoothing (x-axis) and other methods (y-axis), including GLM with FWHM=6 Gaussian smoothing (**a**; S6GLM), conventional CCA **(b)**, CCA with a sum constraint (**c**; sumCCA), and the proposed DNN model **(d)**. The bins above the diagonal line indicated that correlation obtained from the other method was greater than the correlation obtained from GLM (>r_GLM); and the bins under the diagonal line indicated that the correlation obtained from the other method was less than the correlation obtained from GLM (>r_GLM). The color represented the number of voxels in each bin.

The 2D histograms of the correlation map from GLM and the other methods are shown in Figure 4, with the color representing the number of voxels in each bin. The bins above the diagonal line indicated that the method on the y-axis had a higher correlation than the method on the x-axis, and the bins below the diagonal line indicated that the method on the y-axis had a lower correlation than the method on the x-axis. Gaussian smoothing consolidated the correlation strength for the active voxels, with the observation of more voxels above the diagonal line. However, a noticeable fraction of inactive voxels in GLM also had higher correlation after Gaussian smoothing (top left corner in Figure 4a). The CCA methods had higher correlations than GLM for nearly all voxels, and the sum constraint alleviated the overestimated correlation for highly inactive voxels (correlation values close to 0). Different from Gaussian smoothing that weakened the correlations for a fraction of voxels (blue oval in Figure 4a), conventional CCA and sumCCA showed a shift toward higher correlation, which was expected since the CCA approaches estimated the spatial smoothing by maximizing the correlation value, and no smoothing was one solution candidate ( $\alpha_{i}=0$ for all neighboring voxels and $\alpha_{i}=1$ for the center voxels). Since the spatial weight coefficient α for certain voxels in conventional CCA did not necessarily satisfy the sum constraint, the spatial filtering with lower correlations was adopted in sumCCA, and thus, the up shift in sumCCA was not as much as in conventional CCA. As to the proposed DNN method, it further restrained the voxels with low correlations in GLM from achieving higher correlations, and most of them were along the diagonal lines in the low correlation range, indicating that DNN did not lead to noticeable over-estimated activation for inactive voxels. Such a discrepancy between DNN and CCA approaches was because of their different cost functions. As for voxels with intermediate to high correlations in GLM, most of them had higher correlations in DNN than in GLM. Collectively, DNN showed an upshift of correlation for voxels that were more likely to be active but did not show a clear shift for voxels that were less likely to be active, which was beneficial for discriminating active voxels from inactive voxels.

**Figure 5:**
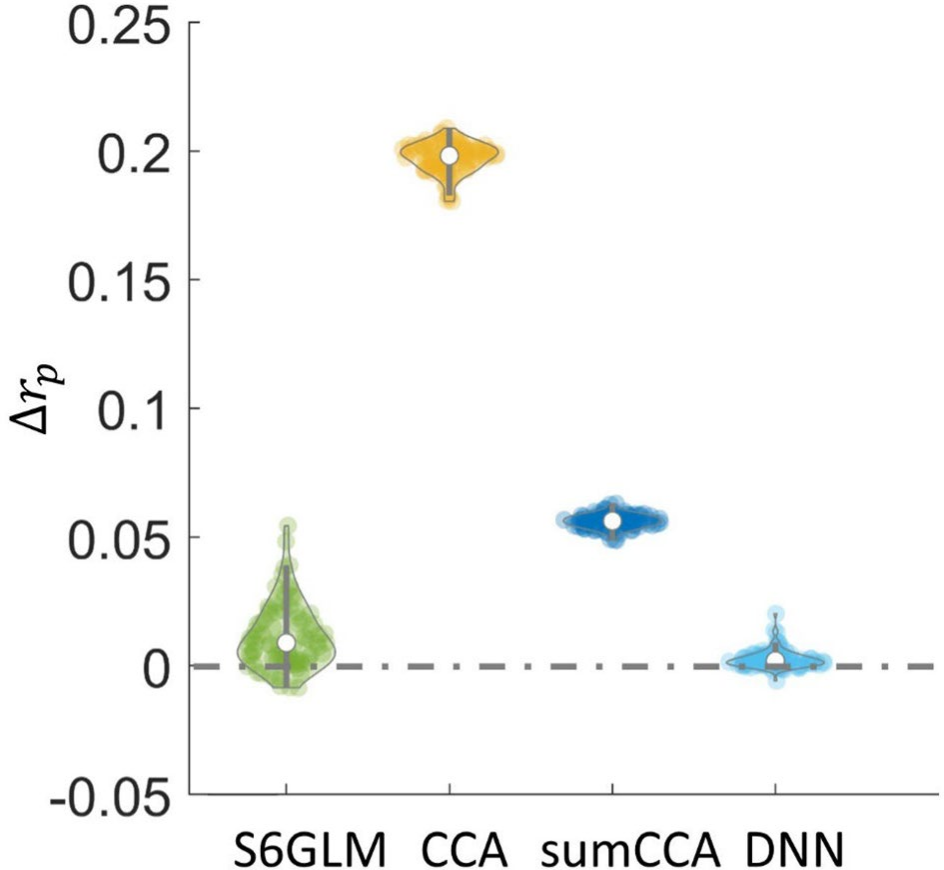
Difference in $r_{p}$ values between other methods and GLM. Wavelet-resampled resting-state fMRI data were generated as the null data for each subject, and then the correlation values were computed with each method. The correlation value at the 99.9 percentile from the null data was denoted as $r_{p}$ . The $\Delta r_{p}$ value was calculated as the difference of $r_{p}$ from one method compared to GLM, which reflected how strongly a method exaggerated the correlation of inactive voxels compared to GLM without spatial smoothing.

With wavelet-resampled null data, the correlation values at the 99.9 percentile, namely $r_{p}$ , were computed for all analysis methods. Figure 5 shows the difference of $r_{p}$ between other analysis methods and GLM without spatial smoothing. CCA without constraint had $r_{p}$ increased by approximately 0.2 compared to GLM. A sum constraint substantially suppressed the difference and had the correlation difference slightly more than 0.05. Gaussian smoothing had the average $r_{p}$ slightly increased with a larger variability between resampled fMRI sessions. In contrast, the proposed DNN model had the $r_{p}$ value negligibly different from GLM with a small variability between resampled fMRI sessions.

**Figure 6:**
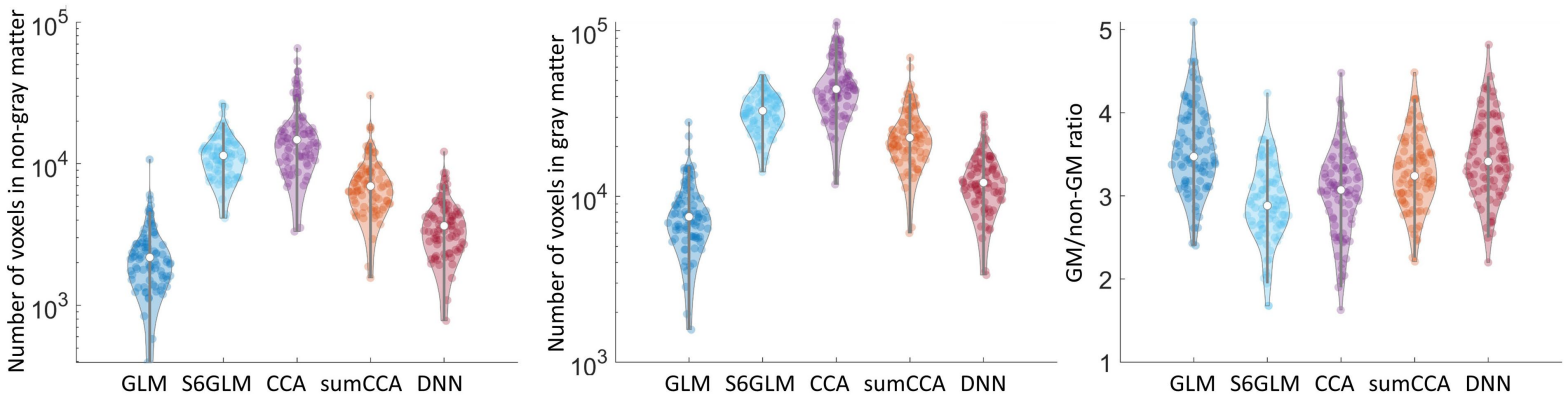
Number of voxels in non-gray matter **(a)** and gray matter tissue **(b)** above the cut-off value $r_{p}$ in each subject. The ratio of voxels between gray matter and non-gray matter was calculated within each subject and is shown in **(c)**.

**Figure 7:**
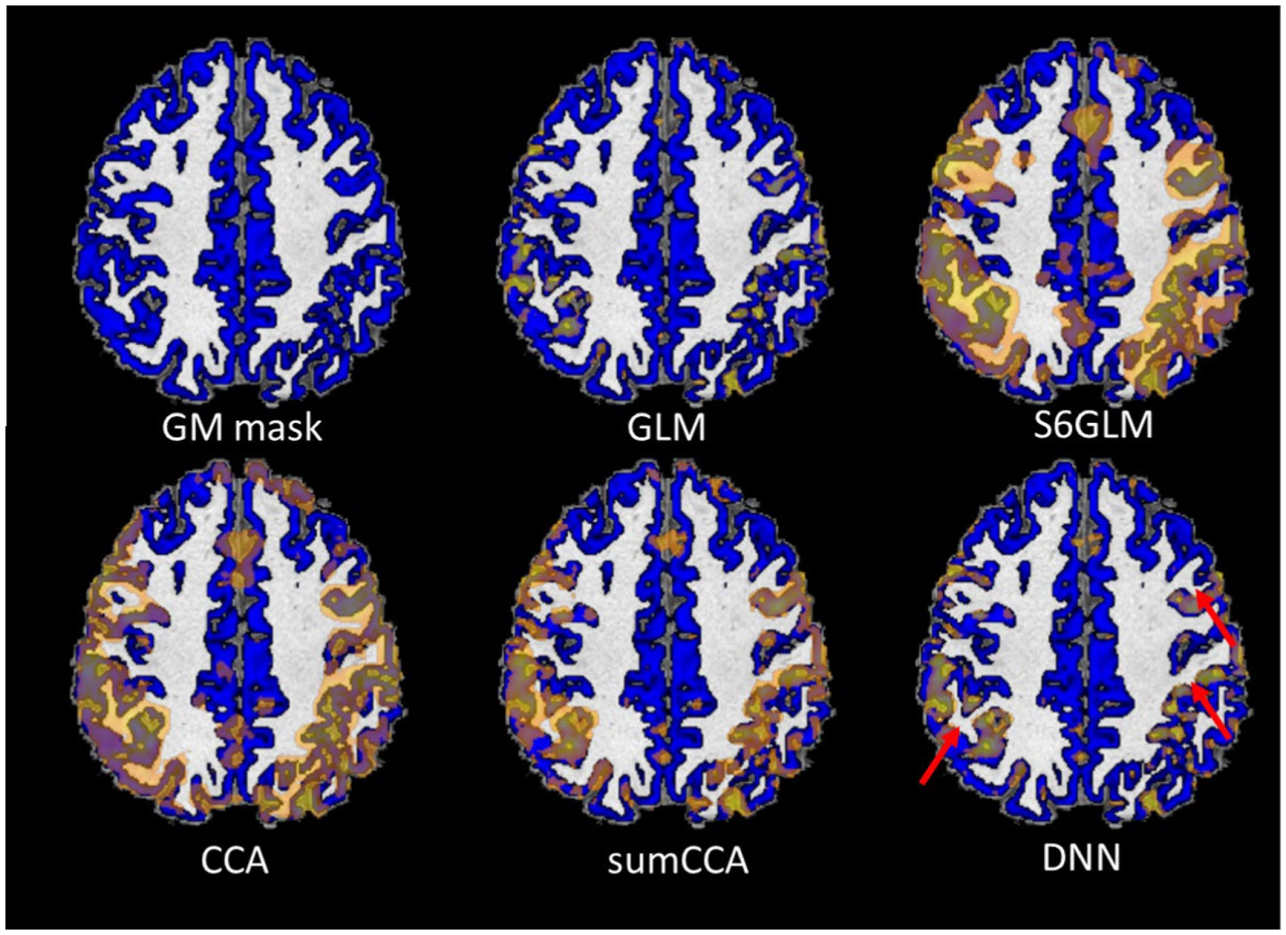
Active map with the cutoff value $r_{p}$ from a single subject. Gray matter mask is overlaid in the image as marked in blue. The areas showing a substantial reduction of bleeding artifacts are marked by red arrows.

We then counted the number of voxels correlating the cutoff value $r_{p}$ within gray matter or non-gray matter. GLM, as an approach without spatial smoothing, had the least number of voxels within gray matter or non-gray matter tissue (Figures 6a,b). All other methods had more voxels above the cut-off value for both gray matter and non-gray matter. We then computed the ratio of the number of voxels between gray matter and non-gray matter. Gaussian smoothing with FWHM = 6 mm had the lowest ratio (Figure 6c). CCA had a higher GM/non-GM ratio, and the value was further improved by implementing a sum constraint on neighboring voxels. The proposed DNN model had the highest GM/non-GM ratio compared to the Gaussian smoothing and CCA approaches, and its value was comparable to GLM without spatial smoothing. The activation map above the cutoff value $r_{p}$ (orange color) overlaid on the gray matter mask (blue color) from a single subject is shown in Figure 7. As marked by red arrows, DNN alleviated the blurring artifact as in traditional spatial smoothing approaches.

## Discussion

In this study, we introduced a DNN-based adaptive smoothing framework for task fMRI data. The proposed DNN framework inherits the flexibility of the local CCA family (Zhuang et al., 2017) in implementing spatial constraints on local neighboring voxels; at the same time, it provides a time-efficient way to consider more neighboring voxels during the process of determining optimal parameters for spatial smoothing. We have applied this technique, together with previously developed methods, to simulated and real task fMRI data, demonstrating its feasibility and robustness for task fMRI activation analysis.

As the ancestor of the proposed DNN method, sumCCA shares some similarities with the DNN method. First, both methods are adaptive spatial smoothing approaches without pre-specified spatial filters. Second, a constraint can be applied on neighboring voxels to alleviate the spatial blurring artifact. Besides the sum constraint, other constraints used in CCA, including the non-negative constraint and the dominance constraint (Cordes et al., 2012), can also be easily implemented in the DNN framework. Third, without considering computational efficiency, both methods are ideally applicable to various fMRI acquisition schemes. For example, for data acquired with fine in-slice resolution but large slice thickness (e.g., 1 mm × 1 mm × 5 mm), in-slice 3 × 3 neighboring voxels, or even more in-slice neighboring voxels, might be preferred over including voxels from neighboring slices (e.g., 3 × 3 × 3 neighboring voxels). To restrict the DNN to in-slice neighboring voxels, the 3 × 3 × 3 convolutional filters can be replaced with 3 × 3 × 1 filters, while the rest of the algorithm remains unchanged for analysis.

At the same time, the proposed DNN method is distinct from traditional CCA methods in multiple aspects. First, different from sumCCA, which is carried out at each voxel independently and repetitively across the entire brain, DNN takes advantage of the convolutional layers to derive a set of filters from the data itself for all the voxels and then used the fully connected layers to optimize the weights of the output time series from the convolutional layers. Including more neighboring voxels can be achieved by stacking additional convolutional layers or increasing the window size of the filters, which is much more computationally efficient compared to sumCCA, whose computational time substantially increases with the number of neighboring voxels included in the analysis (Yang et al., 2018). Second, these two methods have different criteria to optimize the spatial smoothing parameters. The contribution of neighboring voxels in sumCCA is optimized to maximize the correlation between filtered time series and task design matrix regardless of the anatomical information. The correlation between the filtered time series and task design matrix is also computed in the DNN framework, but instead of maximizing the correlation for all voxels, it maximizes the ratio of the mean correlation between gray matter and non-gray matter tissue. The task-related BOLD activity is expected to be mainly, if not all, located in gray matter. When spatial blurring artifacts occur, the BOLD signal in the non-gray matter tissue surrounding active gray matter voxels, as assessed by correlation, is artificially increased. A spatial smoothing strategy that suppresses the noise in gray matter, leading to stronger correlation and having no or less impact on non-gray matter tissue, is preferred. For this reason, the ratio, instead of the correlation itself, is used for optimizing the DNN model. Third, unlike sumCCA requires non-parametric tests for the statistical analysis, parametric statistical tests are suggested to be applicable for the DNN method. It is found that the correlation at 99.9 percentile, namely $r_{p}$ , from resampled data after DNN has a negligible deviation from the resampled data without any spatial smoothing, in contrast to a substantial increase as observed with CCA methods. This finding suggests that the null distribution without spatial smoothing well approximates the null distribution after DNN spatial smoothing; therefore, the parametric statistical tests used in GLM are applicable to DNN-processed data, which is more time efficient than non-parametric tests.

Utilizing anatomical information to assist with fMRI smoothing is not exclusive to the DNN method. With the assistance of structural MRI data, 3D volumetric fMRI data can be first projected to the surface of the brain, and smoothing is carried out with the surface-based data. This approach can help reduce signal blurring between different cortical areas (e.g., across sulcal banks) (Brodoehl et al., 2020); however, it is limited by its reliance on precise anatomical registration and accurate projection of volumetric fMRI data onto the cortical surface (Tucholka et al., 2012). FMRI data contain artifacts due to geometric distortion in the EPI sequence, which are not fully corrected during preprocessing (Hutton et al., 2002). In addition, the typical resolution of fMRI data makes it challenging to project volumetric fMRI data accurately on the cortical surface due to the thin cortical thickness and the close proximity of opposing banks within a sulcus (Operto et al., 2008). In laminar fMRI research, where the dependence of activity on cortical depth is of particular interest, the boundary between gray matter and white matter or cerebrospinal fluid is used as the basis to delineate cortical layers. Spatial smoothing in laminar fMRI studies takes advantage of the anatomical information of cortical layers to smooth data within each cortical layer (Huber et al., 2017) or across a few neighboring layers (Blazejewska et al., 2019). The spatial smoothing used in surface-based fMRI and laminar fMRI is purely based on anatomical information, and the same smoothing parameter is used for all vertices. These factors make the signal blurring between neighboring vertices inevitable. In contrast, the DNN method is a volume-based smoothing strategy with spatial smoothing parameters depending on the functional signal difference between brain tissues, which adaptively optimizes smoothing for each voxel. Although anatomical information is also required in DNN, erosion is used in defining the non-GM mask for computing the customized cost function, which reduces the risk of GM voxels being included. A small fraction of voxel misclassification is unlikely to have a fundamental effect on the model. In addition, DNN does not involve the process of relying on anatomical structure for data interpolation. Developing a method to integrate the information of cortical layers/surface and the signal difference between brain tissues would be a future direction of the study.

Despite being fundamentally different from the DNN method, various non-smoothing-based methods can, in principle, be used to suppress noise components in fMRI time series, including temporal autocorrelation correction (Olszowy et al., 2019), motion correction (Zaitsev et al., 2017), and ICA/PCA-based or deep learning-based denoising strategies (Pruim et al., 2015; Vizioli et al., 2021; Yang et al., 2020b). These approaches can be used together with DNN. Performing a thorough comparison under all possible combinations of these methods is beyond the scope of this study. The primary aim of this study is to introduce a flexible adaptive smoothing framework for task fMRI.

There are a few limitations to the study. First, the DNN method assumes that the task-related neuronal activity is well-modeled in the design matrix. A canonical hemodynamic response function was used in the study to model neuronal response after the stimuli. Previous studies demonstrated the variability of the hemodynamic response function between subjects or even between regions (Handwerker et al., 2004; Aguirre et al., 1998). Inspecting the result from traditional methods such as GLM with/without Gaussian smoothing to evaluate if the task-related neuronal response is well captured with canonical hemodynamic response function before applying DNN is recommended. Instead of using a canonical hemodynamic response function, deriving a subject-specific hemodynamic response function to model task response could be an alternative approach (Lindquist et al., 2009). Second, as a deep learning-based approach, the proposed DNN approach requires a large number of voxels to train the model. When fMRI data is acquired with partial brain coverage or with a coarse resolution, its feasibility needs to be examined before extensively applying DNN for analysis. Third, the spatial smoothing is optimized based on the correlation of time series with task design matrix between gray matter and non-gray matter tissues. Therefore, the activity for the task conditions not modeled in the design matrix might be suboptimal. As for the resting-state fMRI data, the task design matrix can possibly be replaced by the time series from given seed regions. However, because the time series from the seed regions is contaminated by various noise, using it to train the DNN model might be detrimental instead of being beneficial for the follow-up statistical analysis. Future research is warranted to investigate the potential utility of this approach on resting-state fMRI data.

In summary, an efficient DNN-based adaptive spatial smoothing strategy is proposed for task fMRI activation analysis. Stacking multiple convolutional layers can efficiently consider more neighboring voxels during the adaptive spatial smoothing process, which overcomes the limitation of computational burden in CCA approaches. At the same time, a family of constraints used in CCA can be easily implemented in the proposed method, which makes the approach highly flexible and can be revised for various fMRI acquisition schemes. Furthermore, the novel cost function introduced in the DNN method does not exaggerate the activation of inactive voxels, which suggests that the parametric statistical test used for GLM can be used to determine the statistical significance of brain activation derived from DNN-smoothed time series.

## Data Availability

Publicly available datasets were analyzed in this study. This data can be found at: https://www.humanconnectome.org/study/hcp-young-adult/document/1200-subjects-data-release.

## References

[ref1] Aguirre G. K. Zarahn E. D'Esposito M. (1998). The variability of human, Bold hemodynamic responses. NeuroImage 8, 360–369. doi: 10.1006/nimg.1998.0369, PMID: 9811554

[ref2] Arthurs O. J. Boniface S. (2002). How well do we understand the neural origins of the fmri Bold signal? Trends Neurosci. 25, 27–31. doi: 10.1016/S0166-2236(00)01995-0, PMID: 11801335

[ref3] Barch D. M. Burgess G. C. Harms M. P. Petersen S. E. Schlaggar B. L. Corbetta M.. (2013). Function in the human connectome: task-fmri and individual differences in behavior. NeuroImage 80, 169–189. doi: 10.1016/j.neuroimage.2013.05.033, PMID: 23684877 PMC4011498

[ref4] Bizzi A. Blasi V. Falini A. Ferroli P. Cadioli M. Danesi U.. (2008). Presurgical functional Mr imaging of language and motor functions: validation with intraoperative electrocortical mapping. Radiology 248, 579–589. doi: 10.1148/radiol.2482071214, PMID: 18539893

[ref5] Blazejewska A. I. Fischl B. Wald L. L. Polimeni J. R. (2019). Intracortical smoothing of small-voxel fmri data can provide increased detection power without spatial resolution losses compared to conventional large-voxel fmri data. NeuroImage 189, 601–614. doi: 10.1016/j.neuroimage.2019.01.054, PMID: 30690157 PMC6668026

[ref6] Brodoehl S. Gaser C. Dahnke R. Witte O. W. Klingner C. M. (2020). Surface-based analysis increases the specificity of cortical activation patterns and connectivity results. Sci. Rep. 10:5737. doi: 10.1038/s41598-020-62832-z, PMID: 32235885 PMC7109138

[ref7] Cordes D. Jin M. Curran T. Nandy R. (2012). Optimizing the performance of local canonical correlation analysis in fmri using spatial constraints. Hum. Brain Mapp. 33, 2611–2626. doi: 10.1002/hbm.21388, PMID: 23074078 PMC5551496

[ref8] Courtemanche M. J. Sparrey C. J. Song X. MacKay A. D'Arcy R. C. N. (2018). Detecting white matter activity using conventional 3 tesla fmri: an evaluation of standard field strength and hemodynamic response function. NeuroImage 169, 145–150. doi: 10.1016/j.neuroimage.2017.12.008, PMID: 29229580

[ref9] Finn E. S. Shen X. Scheinost D. Rosenberg M. D. Huang J. Chun M. M.. (2015). Functional connectome fingerprinting: identifying individuals using patterns of brain connectivity. Nat. Neurosci. 18, 1664–1671. doi: 10.1038/nn.4135, PMID: 26457551 PMC5008686

[ref10] Friman O. Borga M. Lundberg P. Knutsson H. (2003). Adaptive analysis of fmri data. NeuroImage 19, 837–845. doi: 10.1016/s1053-8119(03)00077-6, PMID: 12880812

[ref11] Friman O. Cedefamn J. Lundberg P. Borga M. Knutsson H. (2001). Detection of neural activity in functional Mri using canonical correlation analysis. Magn. Reson. Med. 45, 323–330. doi: 10.1002/1522-2594(200102)45:2<323::AID-MRM1041>3.0.CO;2-#, PMID: 11180440

[ref12] Garcia-Garcia A. Orts-Escolano S. Oprea S. Villena-Martinez V. Garcia-Rodriguez J. (2017). A review on deep learning techniques applied to semantic segmentation. arXiv preprint arXiv:1704.06857.

[ref13] Glasser M. F. Sotiropoulos S. N. Wilson J. A. Coalson T. S. Fischl B. Andersson J. L.. (2013). The minimal preprocessing pipelines for the human connectome project. NeuroImage 80, 105–124. doi: 10.1016/j.neuroimage.2013.04.127, PMID: 23668970 PMC3720813

[ref14] Handwerker D. A. Ollinger J. M. D'Esposito M. (2004). Variation of Bold hemodynamic responses across subjects and brain regions and their effects on statistical analyses. NeuroImage 21, 1639–1651. doi: 10.1016/j.neuroimage.2003.11.029, PMID: 15050587

[ref15] Hartvig N. V. Jensen J. L. (2000). Spatial mixture modeling of fmri data. Hum. Brain Mapp. 11, 233–248. doi: 10.1002/1097-0193(200012)11:4<233::aid-hbm10>3.0.co;2-f, PMID: 11144753 PMC6871941

[ref16] Huber L. Handwerker D. A. Jangraw D. C. Chen G. Hall A. Stüber C.. (2017). High-resolution Cbv-fmri allows mapping of laminar activity and connectivity of cortical input and output in human M1. Neuron 96, 1253–1263.e7. e7. doi: 10.1016/j.neuron.2017.11.005, PMID: 29224727 PMC5739950

[ref17] Huber L. Finn E. S. Handwerker D. A. Bönstrup M. Glen D. R. Kashyap S.. (2020). Sub-millimeter fmri reveals multiple topographical digit representations that form action maps in human motor cortex. NeuroImage 208:116463. doi: 10.1016/j.neuroimage.2019.116463, PMID: 31862526 PMC11829252

[ref18] Huettel S.A. Song A.W. McCarthy G., (2004). Functional magnetic resonance imaging.

[ref19] Hutton C. Bork A. Josephs O. Deichmann R. Ashburner J. Turner R. (2002). Image distortion correction in fmri: a quantitative evaluation. NeuroImage 16, 217–240. doi: 10.1006/nimg.2001.1054, PMID: 11969330

[ref20] Kamitani Y. Sawahata Y. (2010). Spatial smoothing hurts localization but not information: pitfalls for brain mappers. NeuroImage 49, 1949–1952. doi: 10.1016/j.neuroimage.2009.06.040, PMID: 19559797

[ref21] Lawrence S. J. D. Formisano E. Muckli L. de Lange F. P. (2019). Laminar fmri: Applications for cognitive neuroscience. NeuroImage 197, 785–791. doi: 10.1016/j.neuroimage.2017.07.004, PMID: 28687519

[ref22] Li M. Newton A. T. Anderson A. W. Ding Z. Gore J. C. (2019). Characterization of the hemodynamic response function in white matter tracts for event-related fmri. Nat. Commun. 10:1140. doi: 10.1038/s41467-019-09076-2, PMID: 30850610 PMC6408456

[ref23] Lindquist M. A. Meng Loh J. Atlas L. Y. Wager T. D. (2009). Modeling the hemodynamic response function in fmri: efficiency, bias and mis-modeling. NeuroImage 45, S187–S198. doi: 10.1016/j.neuroimage.2008.10.065, PMID: 19084070 PMC3318970

[ref24] Liu T. T. (2016). Noise contributions to the fmri signal: an overview. NeuroImage 143, 141–151. doi: 10.1016/j.neuroimage.2016.09.008, PMID: 27612646

[ref25] Olszowy W. Aston J. Rua C. Williams G. B. (2019). Accurate autocorrelation modeling substantially improves fmri reliability. Nat. Commun. 10:1220. doi: 10.1038/s41467-019-09230-w, PMID: 30899012 PMC6428826

[ref26] Operto G. Bulot R. Anton J. L. Coulon O. (2008). Projection of fmri data onto the cortical surface using anatomically-informed convolution kernels. NeuroImage 39, 127–135. doi: 10.1016/j.neuroimage.2007.08.039, PMID: 17931891

[ref27] Pruim R. H. Mennes M. van Rooij D. Llera A. Buitelaar J. K. Beckmann C. F. (2015). Ica-aroma: a robust Ica-based strategy for removing motion artifacts from fmri data. NeuroImage 112, 267–277. doi: 10.1016/j.neuroimage.2015.02.064, PMID: 25770991

[ref28] Razzak M. I. Naz S. Zaib A. (2018). “Deep learning for medical image processing: overview, challenges and the future,” in Classification in BioApps. Cham: Springer, 323–350. doi: 10.1007/978-3-319-65981-7_12

[ref29] Rydell J. Knutsson H. Borga M. (2006). On rotational invariance in adaptive spatial filtering of fmri data. NeuroImage 30, 144–150. doi: 10.1016/j.neuroimage.2005.09.002, PMID: 16257235

[ref30] Schilling K. G. Li M. Rheault F. Gao Y. Cai L. Zhao Y.. (2023). Whole-brain, gray, and white matter time-locked functional signal changes with simple tasks and model-free analysis. Proc. Natl. Acad. Sci. 120:e2219666120. doi: 10.1073/pnas.2219666120, PMID: 37824529 PMC10589709

[ref31] Signorelli F. Guyotat J. Isnard J. Schneider F. Mohammedi R. Bret P. (2001). The value of cortical stimulation applied to the surgery of malignant gliomas in language areas. Neurol. Sci. 22, 3–10. doi: 10.1007/s100720170029, PMID: 11487189

[ref32] Silva M. A. See A. P. Essayed W. I. Golby A. J. Tie Y. (2018). Challenges and techniques for presurgical brain mapping with functional Mri. NeuroImage Clin. 17, 794–803. doi: 10.1016/j.nicl.2017.12.008, PMID: 29270359 PMC5735325

[ref33] Tanabe J. Miller D. Tregellas J. Freedman R. Meyer F. G. (2002). Comparison of detrending methods for optimal fmri preprocessing. NeuroImage 15, 902–907. doi: 10.1006/nimg.2002.1053, PMID: 11906230

[ref34] Thompson B. (2005). “Canonical correlation analysis,” in Encyclopedia of statistics in behavioral science. eds. Everitt B. S. Howell D. C.. doi: 10.1002/0470013192.bsa068

[ref35] Tucholka A. Fritsch V. Poline J. B. Thirion B. (2012). An empirical comparison of surface-based and volume-based group studies in neuroimaging. NeuroImage 63, 1443–1453. doi: 10.1016/j.neuroimage.2012.06.019, PMID: 22732555

[ref36] Tzourio-Mazoyer N. Landeau B. Papathanassiou D. Crivello F. Etard O. Delcroix N.. (2002). Automated anatomical labeling of activations in Spm using a macroscopic anatomical parcellation of the Mni Mri single-subject brain. NeuroImage 15, 273–289. doi: 10.1006/nimg.2001.0978, PMID: 11771995

[ref37] Vizioli L. Moeller S. Dowdle L. Akçakaya M. de Martino F. Yacoub E.. (2021). Lowering the thermal noise barrier in functional brain mapping with magnetic resonance imaging. Nat. Commun. 12:5181. doi: 10.1038/s41467-021-25431-8, PMID: 34462435 PMC8405721

[ref38] Yang Z. Zhuang X. Mishra V. Sreenivasan K. Cordes D. (2020a). Cast: a multi-scale convolutional neural network based automated hippocampal subfield segmentation toolbox. NeuroImage 218:116947. doi: 10.1016/j.neuroimage.2020.116947, PMID: 32474081 PMC7442241

[ref39] Yang Z. Zhuang X. Sreenivasan K. Mishra V. Curran T. Byrd R.. (2018). 3D spatially-adaptive canonical correlation analysis: local and global methods. NeuroImage 169, 240–255. doi: 10.1016/j.neuroimage.2017.12.025, PMID: 29248697 PMC5856611

[ref40] Yang Z. Zhuang X. Sreenivasan K. Mishra V. Curran T. Cordes D. (2020b). A robust deep neural network for denoising task-based fmri data: an application to working memory and episodic memory. Med. Image Anal. 60:101622. doi: 10.1016/j.media.2019.101622, PMID: 31811979 PMC6980789

[ref41] Yassa M. A. Stark C. E. (2011). Pattern separation in the hippocampus. Trends Neurosci. 34, 515–525. doi: 10.1016/j.tins.2011.06.006, PMID: 21788086 PMC3183227

[ref42] Zaitsev M. Akin B. LeVan P. Knowles B. R. (2017). Prospective motion correction in functional Mri. NeuroImage 154, 33–42. doi: 10.1016/j.neuroimage.2016.11.014, PMID: 27845256 PMC5427003

[ref43] Zhuang X. Yang Z. Cordes D. (2020). A technical review of canonical correlation analysis for neuroscience applications. Hum. Brain Mapp. 41, 3807–3833. doi: 10.1002/hbm.25090, PMID: 32592530 PMC7416047

[ref44] Zhuang X. Yang Z. Curran T. Byrd R. Nandy R. Cordes D. (2017). A family of locally constrained Cca models for detecting activation patterns in fmri. NeuroImage 149, 63–84. doi: 10.1016/j.neuroimage.2016.12.081, PMID: 28041980 PMC5493994

